# Menthol in Diphtheria

**Published:** 1890-11-15

**Authors:** 


### MENTHOL IN DIPHTHERIA.

There can be no doubt that diphtheria, though ultimately a general, isprimarily a local disease; we mean that while in scarlatina the sore throat is, like the rash, one evidence of the general condition, in diphtheria the commencement of the morbid appearances in the pharynx, larynx, or nares indicates the seat of the inoculation where the bacilli grow and multiply, subsequently infecting the organism with their secretions, and that by early local treatment with germicides the general poisoning may be minimised. We gave some time since an account of Mr. Curgenven's employment of sprays of oil of Eucalyptus, or Tucker's Eucalyptus antiseptic, which consists of this oil with the addition of Menthol, Thymol, &c.; and we have recently noticed that Hahn and Ziegler recommend the use of menthol, the latter employing it in solution, the former preferring it as a powder, rubbing it up with sugar in the proportion of 1 in 10 or 20, applied with a brush, as much of the membrane as possible being brushed off, so that the menthol, which in the dry state adheres better than it does in solution, may come into actual contact with the underlying surface. These oils are powerful germicides and far less irritating to the living tissues than liq. sod. chlorinat., liq. ferri perchl., or hydrochloric acid.

